# Associations between language, telehealth, and clinical outcomes in patients with cancer during the COVID‐19 pandemic

**DOI:** 10.1002/cam4.70099

**Published:** 2024-09-23

**Authors:** Armon Azizi, Aditya Mahadevan, Jagmeet S. Arora, Elaine Chiao, Sora Tanjasiri, Farshid Dayyani

**Affiliations:** ^1^ School of Medicine University of California Irvine Irvine California USA; ^2^ Department of Health, Society and Behavior, Program of Public Health University of California Irvine Irvine California USA; ^3^ Division of Hematology/Oncology University of California Irvine Health Orange California USA

**Keywords:** cancer, COVID‐19, health disparities, language barrier, telehealth

## Abstract

**Background:**

The COVID‐19 pandemic prompted a surge in telehealth utilization. However, language barriers have emerged as a potential obstacle to effective telemedicine engagement, impacting millions of limited English proficient (LEP) individuals. Understanding the role of language spoken in telehealth outcomes is critical, particularly in cancer care, in which consistent follow‐up and communication are vital. The primary objective was to assess the impact of telehealth utilization and primary language spoken on clinical outcomes in cancer patients.

**Methods:**

This study utilized a retrospective cohort design, encompassing cancer patients seen at the Chao Family Comprehensive Cancer Center between March 1, 2020, and December 31, 2022. The study incorporated both in‐person and telehealth visits, examining the association between encounter type and clinical outcomes.

**Results:**

The study included 7890 patients with more than one outpatient visit during the study period. There was decreased telehealth utilization in non‐English speaking cancer patients throughout the pandemic. Increased telehealth utilization was associated with higher rates of admission, irrespective of cancer type. Additionally, telehealth visits were associated with longer duration of subsequent admissions compared to in‐person visits. Spanish‐speaking patients utilizing telehealth had higher rates of re‐admission compared to English speakers utilizing telehealth. Patients who died had higher rates of telehealth utilization compared to patients who survived.

**Conclusions and Relevance:**

This study demonstrates that primary language spoken is associated with differences in telehealth utilization and associated outcomes in cancer patients. These differences suggest that the interplay of telehealth and language could contribute to widening of disparities in clinical outcomes in these populations. The study underscores the need to optimize telehealth usage and minimize its limitations to enhance the quality of cancer care in a telehealth‐driven era.

## Graphical abstract

This study investigated the impact of telehealth and primary language on cancer patient outcomes, using a retrospective cohort design involving 7890 patients at the Chao Family Comprehensive Cancer Center from March 1, 2020, to December 31, 2022. Results indicated reduced telehealth usage among non‐English speakers during the pandemic, with increased telehealth linked to higher admission rates regardless of cancer type and stage, and longer subsequent admissions compared to in‐person visits. Furthermore, Spanish‐speaking patients using telehealth had higher re‐admission rates, and deceased patients exhibited greater telehealth utilization compared to survivors.

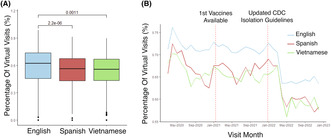

## 1 INTRODUCTION

Telehealth has been employed extensively in the field of oncology, in which patient outcomes are often contingent on consistent follow‐up visits and effective communication.^1^ Defined as patient care performed via a remote electronic interface, virtual telehealth patient visits provide patients an opportunity for accessible care without the need for travel.^2^ Prior studies involving cancer patients have found disparities with regard to telehealth utilization and outcomes, implicating race, zip code, and insurance status as predictors of poor outcomes.^3^, ^4^, ^5^ Limited English proficiency (LEP) is a growing challenge within clinical settings in the United States, affecting over 2.5 million patients nationally.^6^, ^7^ Previous studies have linked LEP to poor patient outcomes including increased rates of hospital stay, in‐hospital mortality, and readmission.^8^, ^9^, ^10^, ^11^ While prior studies have found that LEP is associated with lower rates of telehealth engagement, there is little to no data investigating whether telehealth‐related disparities resulting from LEP affect clinical outcomes in patients with cancer.^12^, ^13^ Furthermore, while patients with cancer have demonstrated satisfaction with telehealth‐based care, the direct impact of telehealth utilization on care quality and clinical outcomes has not been widely investigated, despite a demonstrated need.^14^, ^15^, ^16^, ^17^

Although the COVID‐19 pandemic is no longer considered a national emergency, telehealth remains a highly valued, integral aspect of patient care in several fields.^18^, ^19^, ^20^ Hence, it is vital to investigate the effects of telehealth usage on clinical outcomes. Since prior evidence indicates that telehealth usage and clinical outcomes vary by patient characteristics, it is vital to investigate whether the utilization of telehealth by LEP patients has further contributed to disparities in healthcare outcomes secondary to language barriers.^3^, ^4^, ^5^, ^12^, ^21^

This study evaluated the association between telehealth utilization and primary language spoken on clinical outcomes in patients with cancer seen at an NCI‐designated comprehensive cancer center. We hypothesized that LEP patients utilizing telehealth would have differences in usage rates and subsequent clinical outcomes compared to English speakers.

## 2 METHODS

### 2.1 Cohort identification

Prior to data acquisition, ethical approval and a total waiver of written informed consent was granted by the Institutional Review Board (IRB) at UC Irvine (Protocol number: 1910). Patients were identified using the UC Irvine analytics database to include all patients actively seen at the Chao Family Comprehensive Cancer Center (CFCCC) between 3/1/2020 and 12/31/2022. Patients were included if they were ≥18 years of age, had a cancer diagnosis ICD code in their chart, and had greater than one outpatient visit (either telehealth or in‐person) scheduled during the inclusion dates. All methods in the study were conducted in accordance with STROBE guidelines.

### 2.2 Data acquisition

Data from electronic health records was extracted with a query in SQL Server Management Studio from the UC Irvine Observational Medical Outcomes Partnership database using a data standardization process called the Common Data Model. This allows for efficient analysis of medical terms across different domains by accommodating both clinical and claims data from multiple sources. Variables extracted included age, sex, race, ethnicity, cancer diagnosis ICD codes, language spoken, in person visit dates during the study period, telehealth visit dates during the study period, ED visits and durations during the study period, hospital admissions and durations during the study period, and death date (if applicable). Patient data were stored, and all analyses were performed within the UC Irvine PVCE server, a secure HIPPA‐compliant remote server.

In‐person visits were defined as provider‐patient interactions occurring in‐person at the CFCCC. Telehealth (i.e., virtual visits), were defined as any instance of virtual interaction between provider and patient, including phone calls or video visits. Readmission was defined as a repeat admission within 30 days of discharge.

### 2.3 Statistical methods

All analyses were performed in R version 4.2.2 using the tableone, dplyr, and ggplot packages. Two‐sided student's *t*‐tests were used to compare continuous variables between patient groups and Fisher's exact tests were used to compare discrete variables between groups. For correlation analyses, Pearson product–moment correlations were used and, when assessing the association between virtual visit utilization and rates of emergency department visits or hospitalization rate, linear regression was utilized to fit a model to the data. For multivariable analyses, linear regression was performed with the R *lm* function with clinical covariates (e.g., cancer type, stage) included in the model. To control for disease severity and cancer stage, we included cancer type and the presence of distant metastases as coded for in the *International Classification of Diseases*‐10 (C79), in our multivariable analysis.^22^

## 3 RESULTS

### 3.1 Demographics

A total of 7890 patients were identified and included in the study. Our cohort was 49.6% male (*n* = 3916) and 50.4% female (*n* = 3974), with a median age of 64 (range 18–101). The three most spoken languages as determined by preferred language during visit were English (75.4%, *n* = 5951), Spanish (12.3%, *n* = 967), and Vietnamese (5.8%, *n* = 461). These languages were selected as subgroups for subsequent analyses. The most common cancer types were breast (14.3%), skin (12.9%), lung/bronchus (11.1%), prostate (11.1%), and non‐Hodgkin's lymphoma (10.9%) (Table 1). A total of 460,514 encounters were recorded, 67.3% of which were telehealth.

**TABLE 1:** Cohort demographics.

Feature	*n*_total_ = 7890
Age, median (range)	64 (18–101)
Sex (%)	
Male	3916 (49.6)
Female	3974 (50.4)
Race (%)	
White	4638 (58.8)
Asian	1673 (21.2)
Multirace	233 (3.0)
Black or African American	204 (2.6)
Unknown	98 (1.2)
Native Hawaiian or Other Pacific Islander	36 (0.5)
American Indian or Alaska Native	15 (0.2)
Other Race	993 (12.5)
Language (%)	
English	5951 (75.4)
Spanish	967 (12.3)
Vietnamese	461 (5.8)
Korean	171 (2.2)
Chinese	143 (1.8)
Other	197 (2.5)
Cancer type (%)	
Malignant neoplasm of breast	1128 (14.3)
Other malignant neoplasm of skin	1016 (12.9)
Malignant neoplasm of bronchus and lung	879 (11.1)
Malignant neoplasm of prostate	872 (11.1)
Diffuse non‐Hodgkin's lymphoma	857 (10.9)
Malignant neoplasm of colon	653 (8.3)
Malignant neoplasm of bladder	555 (7.0)
Malignant neoplasm of liver and intrahepatic bile ducts	509 (6.5)
Malignant melanoma of skin	480 (6.01)

### 3.2 Non‐English speakers have lower telehealth utilization

**FIGURE 1:**
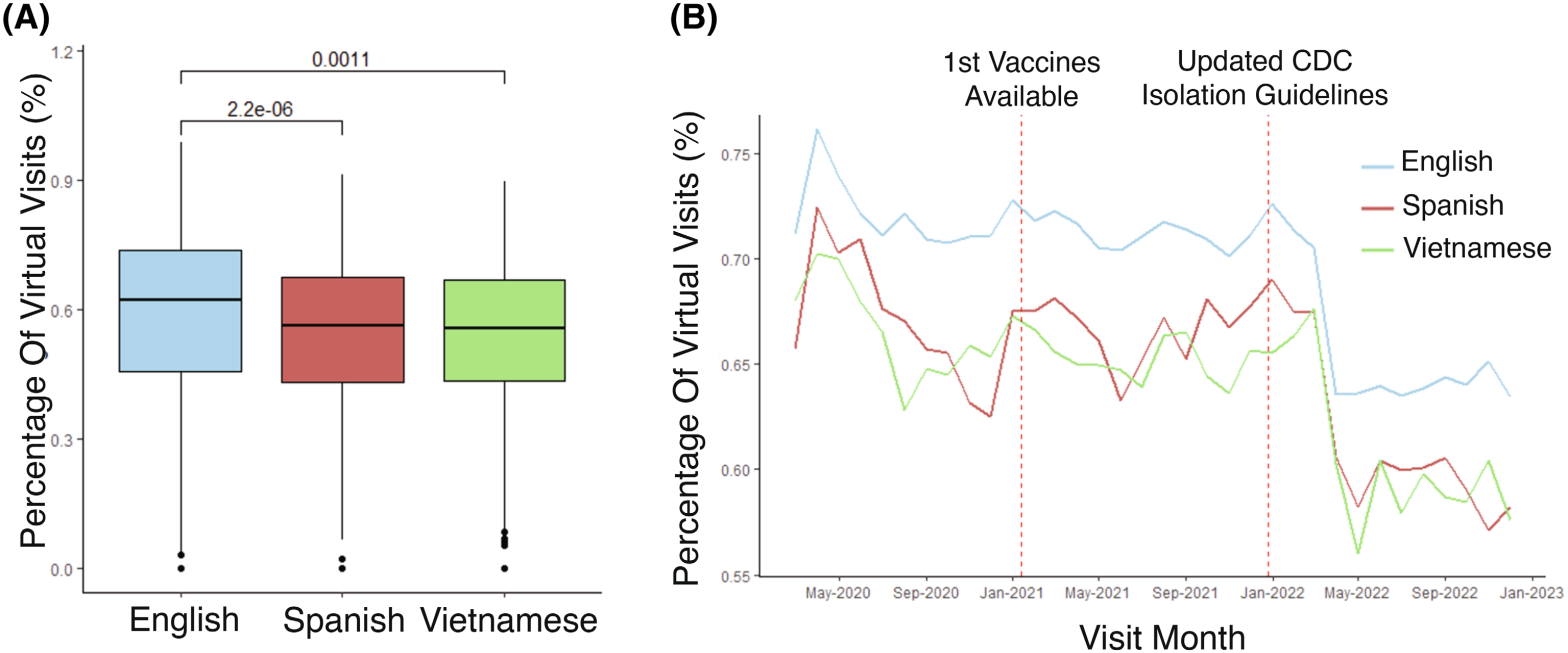
Differences in telehealth utilization in different language groups during the COVID‐19 pandemic. (A) Percentage (%) of telehealth encounters across all patients stratified by primary language spoken. The top three languages from the cohort are shown. Two‐sided student's *t*‐test was performed to compare groups. (B) Percentage (%) of telehealth visits across all patients displayed as a running monthly average across all months included in the study. Each line represents the telehealth encounter rate for either English‐speaking, Spanish‐speaking, or Vietnamese‐speaking patients.

Telehealth visit usage varied by language spoken with non‐English speaking patients utilizing telehealth at significantly lower rates when compared with English‐speaking patients. When examining the median percentage of total encounters that were telehealth across patients, English‐speaking patients had telehealth encounters 62% of the time, Spanish‐speaking patients had telehealth encounters 56% of the time, and Vietnamese‐speaking patients had telehealth encounters 56% of the time (English vs. Spanish *p* < 0.001, English vs. Vietnamese *p* = 0.001, Figure 1A). Telehealth utilization was significantly lower in Spanish‐speaking and Vietnamese‐speaking patients, even when accounting for cancer type and stage, surrogates for disease severity (mean difference in telehealth utilization = −5% for Spanish vs. English and − 5% for Vietnamese vs. English, *p* < 0.001 & *p* < 0.001 respectively, Figure S1).

### 3.3 Telehealth encounter rates are variable over time and associated with vaccination and isolation guidelines

We next examined telehealth utilization over time during the COVID‐19 pandemic by calculating the average fraction of encounters that were telehealth across all patients per month. Telehealth encounters peaked early during the COVID‐19 pandemic in May 2020 and subsequently downtrended, stabilizing by January 2022. Notably, there was a moderate downtrend in telehealth encounters after the first COVID‐19 vaccines became available. There was a larger decrease in telehealth encounters after updated isolation guidelines were released by the CDC in late December 2022 (Figure 1B). Spanish and Vietnamese‐speaking patients had lower telehealth encounter utilization consistently throughout the pandemic with these differences neither increasing nor decreasing over time (Figure 1B).

### 3.4 Telehealth encounters are associated with increased rates of hospitalization

**FIGURE 2:**
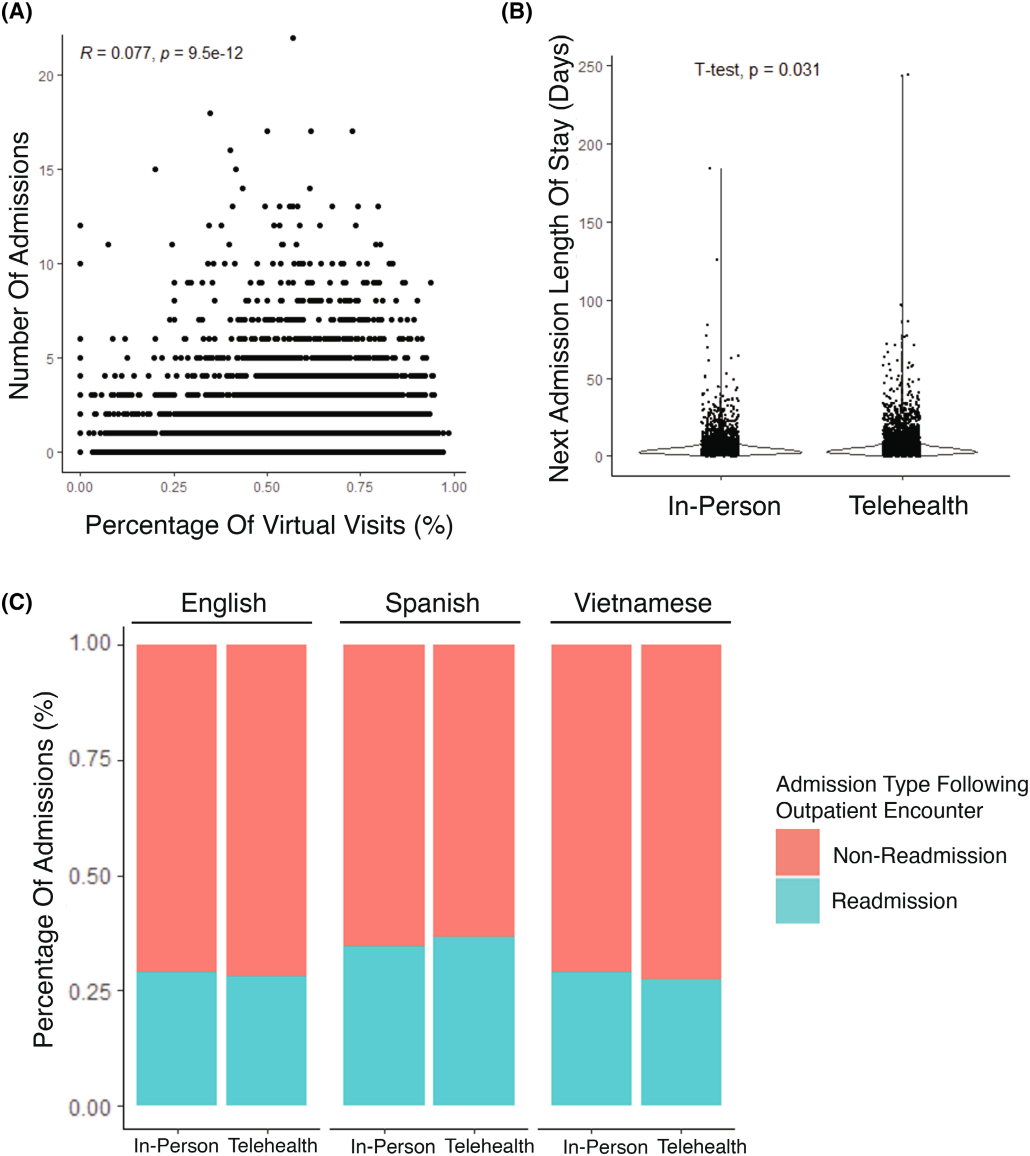
Association between telehealth utilization and admission. (A) Dotplot depicting the association between the percentage (%) of telehealth visits and number of admissions per patient. Each dot represents a single patient in the cohort. (B) Admission duration (days) between admissions following either in‐person or telehealth visits. Mean admission duration 5.8 days versus 6.5 days for admissions following in‐person and telehealth encounters respectively (student's *t*‐test, *p*‐value <0.001). (C) Bar plot showing the fraction of admissions that were re‐admissions (within 30 days of a discharge) following either in‐person or telehealth encounters stratified by language spoken.

To evaluate the association between telehealth encounter rates and clinical outcomes, we first compared the rate of telehealth visits with rates of hospital admission across all patients. Patients with higher rates of telehealth visit usage were more likely to be hospitalized during the study period (*r* = 0.077, *p* < 0.001, Figure 2A), a small but significant association. This association was present at similar levels when patients were isolated by language group. However, the association was not significant in Spanish‐speaking patients (English *r* = 0.088, *p* < 0.001; Vietnamese *r* = 0.093, *p* = 0.045; Spanish *r* = 0.031, *p* = 0.34). Furthermore, telehealth utilization was significantly associated with admission rate when controlling for cancer type using a linear regression model (multivariable regression coefficient of 0.006, *p* < 0.001).

Next, we aimed to determine whether outpatient encounter type (in‐person vs. telehealth) was associated with the duration of subsequent hospital admissions. For each admission, we determined whether the most recent outpatient encounter was in‐person or telehealth and compared the duration of admission between these two groups. The duration of admissions following a telehealth encounter was significantly longer than the duration of admissions following in‐person encounters (telehealth: 6.02 days vs. in‐person: 6.45 days, *p* < 0.05, Figure 2B).

### 3.5 Language spoken is associated with increased rates of re‐admission in patients utilizing telehealth

Hospital re‐admissions are considered an important quality measure and a potentially preventable adverse outcome.^23^ With this mind, we then categorized each admission in our dataset as a re‐admission if the patient had been discharged from the hospital within 30 days of that admission. Re‐admission rate was then defined as the percentage of total admissions that were re‐admissions per patient. There was a small but significant positive association between rates of telehealth visits and re‐admission rates across the entire cohort (*r* = 0.066, *p* < 0.001, Figure S2). Additionally, there was a significant difference in re‐admission rate between English‐speaking and Spanish‐speaking patients (English‐speaking 14.2% vs. Spanish‐speaking: 18.4%, *p* < 0·001) (Figure S3).

We next looked at whether the differences in readmission rate between language groups were affected by the most recent outpatient encounter type. For all hospital admissions, we determined whether the last outpatient visit prior to admission was telehealth or in‐person and subsequently calculated the fraction of hospitalizations that were admissions versus re‐admissions following in‐person versus telehealth encounters. Admissions following telehealth visits were significantly more likely to be readmissions in Spanish‐speaking patients compared to English‐speaking patients (Spanish vs. English‐speaker odds ratio for readmission following telehealth encounters = 1.49 (1.25–1.78), *p* < 0.001) (Figure 2C). Following in‐person visits, there was a smaller, but still significant difference in readmission rates with Spanish‐speakers having higher readmission rates compared to English‐speakers (Spanish vs. English‐speaker odds ratio for readmission following in‐person encounters = 1.29 (1.05–1.59), *p* < 0.05) (Figure 2C). There was no significant difference in readmission rate between admissions following telehealth versus in‐person encounters when examining the whole cohort.

### 3.6 Higher rates of telehealth encounters were observed in oncology patients who died

**FIGURE 3:**
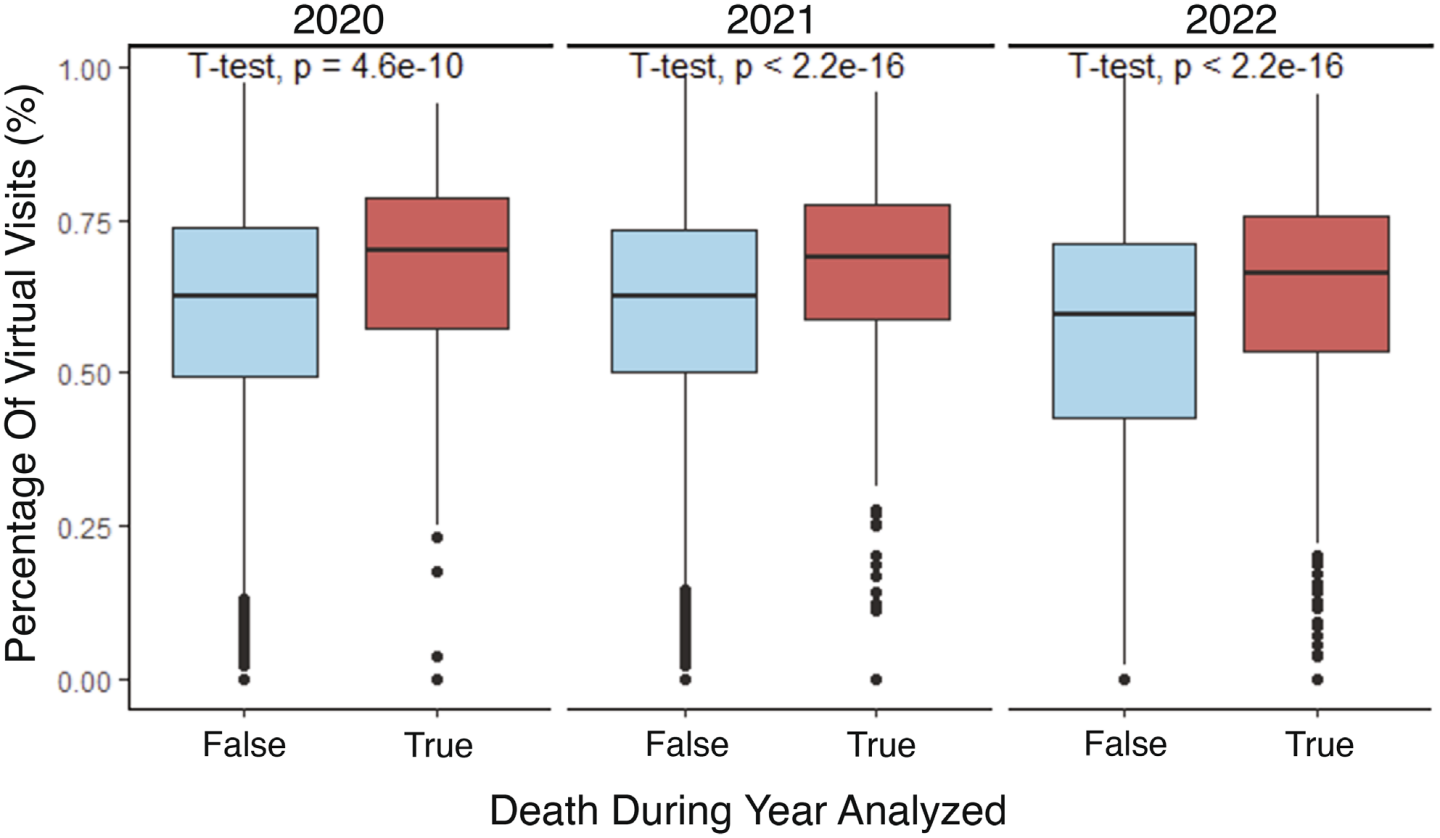
Rates of telehealth encounters between patients that lived versus died during each year of the COVID‐19 pandemic. Patients were only included each year analyzed if they were actively seen (had more than one outpatient visit) during that year.

To determine whether there was an association between mortality and telehealth utilization, we compared the rate of telehealth encounters between patients who lived or died year‐over‐year. Patients were only included in the analysis if they were seen in the outpatient setting that year. Patients who died had significantly higher telehealth encounter rates across all years of the pandemic (% of telehealth visits [lived/died] by year: 2020 [58.7/65.8], 2021 [59.0/66.5], 2022 [53.6/62.6], *p* < 0.001 for all years) (Figure 3). These differences remained and were consistent when patients were stratified by language and cancer stage.

## 4 DISCUSSION

To our knowledge, this is the first study to evaluate the association between language spoken, telehealth utilization, and the clinical implications of telehealth usage in cancer care. We demonstrate that higher rates of telehealth usage were associated with higher numbers of hospital admissions. Furthermore, patient mortality was associated with significantly higher rates of telehealth utilization, irrespective of language spoken or cancer stage. Additionally, telehealth encounters were associated with a longer duration of subsequent admission compared to in‐person visits. LEP patients used telehealth at significantly lower rates than English‐speaking patients, and Spanish‐speaking patients utilizing telehealth experienced significantly higher rates of readmission compared to English‐speakers utilizing telehealth.

### 4.1 Demographics

Our cohort was predominantly English‐speaking and Caucasian. Given that patient characteristics can vary across each geographic region, it is difficult to directly compare our patient population to NCI cancer centers nationally. However, based on prior census data, the ethnolinguistic makeup of our cohort is representative of patients with cancer from Orange County.^24^

### 4.2 Trends in telehealth usage during the pandemic

The initial spike in telehealth usage at the start of the COVID‐19 pandemic, reflected in our data, was consistent with national trends and highlighted the heavy reliance on telehealth early in the pandemic.^25^ The decline of telehealth usage from May 2022 onward was consistent with the declining COVID‐19 case rate in Orange County, gradual transition to in‐person visits, and updates to the isolation guidelines by the CDC and UC Irvine Medical Center.^16^, ^26^ Of note, there were no specific policies implemented which required patients to utilize telehealth at the institution. Patients were never required to utilize telehealth, but it was always offered as an alternative option to in‐person visits. Despite this decline, the stable proportion of telehealth usage from the latter half of 2022 through 2023 suggests that telehealth has become a constant in cancer care. While physicians have voiced a desire to continue to utilize telehealth after the pandemic, our findings are the first to provide concrete, system‐wide evidence that validates these trends in the context of cancer care.^27^ The persistence of telehealth beyond the pandemic makes understanding its impact on care disparities and clinical outcomes even more important, a concern raised in prior investigations.^16^, ^28^

### 4.3 Association between telehealth utilization and rates of hospitalization

Among our patients, increased rates of telehealth utilization were associated with a higher rate of hospitalization. In this analysis, we used the percentage of total visits that were telehealth as opposed to the total number of encounters per patient to correct for confounding factors. We postulated that this association may be explained by differences in prognosis, wherein patients with more severe disease utilize telehealth visits at higher rates while having more frequent admissions. However, when performing analysis of the association between telehealth utilization and admission rates across cancer types and in patients with and without distant metastases, we observed that the association between telehealth and hospitalization was not associated with more advanced disease. This indicates that the positive association between telehealth utilization and hospitalization may not be driven primarily by a specific disease pathology or stage. We hypothesize that an alternative, more plausible explanation is that inherent limitations in patient care during telehealth visits may contribute to increased rates of admission and worse clinical outcomes. To date, there is limited evidence in oncology patients to suggest that telehealth usage may be associated with an increased risk of hospitalization.^29^ While telehealth offers obvious benefits, including increased accessibility and convenience, the inability to perform a physical exam, obtain accurate vitals, and receive real‐time diagnostics is of concern.^30^ Prior investigations of virtual physical exams have shown mixed results and the accuracy of virtual physical exams in patients with cancer is of particular concern.^31^, ^32^ Subtle findings like weight loss or lymphadenopathy may not be readily detected through a virtual interface, a concern shared by clinicians in prior studies.^32^, ^33^ In the context of prior data, our findings suggest that increased reliance on telehealth may contribute to poorer clinical outcomes including increased rates of admission. Further studies are needed to further characterize whether telehealth may contribute to worse clinical outcomes in oncology patients.

Consistent with this assertion, patients whose last visit prior to admission was telehealth had significantly longer lengths of admission compared to patients receiving in‐person visits. Prior studies have hypothesized that the inability to fully evaluate or intervene in chronic illnesses through telehealth visits may lead to clinical deterioration, which in turn could lead to longer admission length.^34^ Our results demonstrate that admissions following telehealth encounters were on average 0·43 days longer than admissions following in‐person encounters. While an increased admission length of approximately half a day may not be clinically significant to individual patients, taken across our entire health system these results suggest that telehealth outpatient visits were associated with an additional 2151 admission days across the 5003 admissions following telehealth visits in our dataset alone. This constitutes a significant impact on physician hours, and hospital and financial resources.

### 4.4 Association between patient mortality and telehealth usage

Patients with cancer who died in 2020, 2021, and 2022 had significantly higher rates of telehealth usage than surviving patients. Early in the pandemic, patients and providers were often forced to use telehealth due to pandemic‐imposed restrictions, causing telehealth usage to skyrocket.^35^ As such, it may follow that COVID‐19 restrictions forced more ill patients to receive telehealth visits, leading to worse outcomes. However, the link between telehealth usage and increased mortality extended well beyond the peak of the COVID‐19 pandemic, suggesting that factors other than COVID‐19 contributed to this association. While mortality in patients with cancer depends on several factors (including age, stage, and performance status), our finding that increased telehealth usage was associated with increased patient mortality is a cause for concern—especially considering that we found positive associations between telehealth usage and measures of poor quality care, specifically higher rates of admission, longer lengths of stay, and higher rates of readmission.^36^

### 4.5 Differences in telehealth utilization and clinical outcomes by language spoken

When comparing telehealth usage by language spoken, we found that Vietnamese and Spanish‐speaking patients had significantly lower rates of telehealth utilization, compared to English‐speaking patients, consistent with prior literature.^37^ Regardless of visit modality, Spanish‐speaking patients are typically readmitted at higher rates than English‐speaking patients, possibly stemming from poor discharge‐related communication, consistent with our data and prior literature.^38^ In addition we found that admissions for Spanish‐speaking patients following telehealth visits were 49% more likely to be readmissions compared to admissions for English‐speakers following telehealth visits. Given that all‐cause readmission rate did not differ significantly by visit modality, the significant differences in readmission rates when stratifying by language spoken inpatients whose prior visit was telehealth suggests that language was a driving factor.

The odds ratio for readmission in Spanish‐speakers versus English‐speakers was higher in admissions following telehealth visits versus in‐person visits. While this difference was not statistically significant, in part due to the smaller sample size of the non‐English speaking groups, these findings suggest that telehealth usage may widen preexisting disparities due to language barriers. While telehealth offers increased access to healthcare for all patients via remote interface, LEP patients face additional challenges in telehealth implementation, particularly due to the incorporation of interpreter services via telephone or videoconference.^39^ Additionally, LEP populations may experience challenges with technology literacy at higher rates, with one study finding that LEP patients were less likely to utilize video features in telehealth visits compared to English speakers.^40^ Socioeconomic status may also influence telehealth equity due to inequities related to health literacy and internet access, which may be exacerbated by learning curves related to telehealth platforms. While frameworks have emerged to ensure equitable telehealth usage and integration in cancer care, limited outcome data exists regarding their usage.^41^ As this is the first study, to our knowledge, to investigate the role of language in cancer patient outcomes following outpatient telehealth visits, these findings warrant further investigation into how telehealth use may contribute to language‐related disparities in care.

Irrespective of language spoken, patients with cancer in our study who died had significantly higher telehealth encounter rates. Though several factors contribute to clinical outcomes, this finding taken with our other findings regarding admission and readmission rate suggests that limitations of the telehealth interface, rather than patients' spoken language, could have contributed to differences in patient mortality. While a prior study found negligible differences in race‐based cancer mortality early in the COVID‐19 pandemic, our study was the first to evaluate the association between language and telehealth usage in cancer patient mortality across the entire pandemic.^42^

### 4.6 Limitations

Our study has a number of limitations. Our data are comprised of patients seen only at a single NCI‐designated comprehensive cancer center in Orange County. In some instances, our analyses were limited by low sample size in some cancer types. Furthermore, the language and telehealth visit data collected from the EMR is based primarily on physician coding. Therefore, the numbers and dates of telehealth visits reflected coding, and may not have captured unrecorded phone calls or other contact between providers and patients. While language preferences are documented directly in the EMR, we are unable to ascertain exact English proficiency based on the recorded preferred language in the EMR. However, recorded preferred language is generally concordant with English proficiency or lack thereof. Additionally, provider‐patient language concordance varies inherently and thus we were unable to control for this when assessing differences between language groups. To address this, we performed chart reviews to confirm that the data collected from the database was accurate and confirmed that telehealth dates were reflective of the visits actually performed. Finally, variables outside the scope of our analysis such as income, literacy, and internet access, may have contributed to our results, but due to the inherent limitations of chart review, these, and other variables that may have contributed to telehealth utilization were not captured in our analysis. However, despite correcting for validated and significant clinical and demographic factors, the persistence of the associations we identified suggests that the interplay between telehealth and language barriers affect clinical outcomes across the diverse array of patients analyzed in this study.

## 5 CONCLUSION

Our study is the first to explore the association between English language‐proficiency and telehealth utilization on clinical outcomes at an NCI‐designated cancer center during the COVID‐19 pandemic. Stable telehealth usage from late 2022 and beyond suggests that telehealth has become a constant in cancer care. However, there were significant associations between telehealth usage and admission rate, admission length, readmission rate, and patient mortality suggesting that the intrinsic limitations of telehealth may play a role in poor patient outcomes. In addition, higher rates of readmission in Spanish‐speaking patients utilizing telehealth suggest that language barriers may play a role in patient readmissions in the context of telehealth usage. While telehealth is an extremely useful tool and can overcome barriers such as distance, transportation, and convenience, its limitations need to be better understood, especially in patients with limited English proficiency. As telehealth is now a mainstay in cancer care, further studies are needed to draw more definitive conclusions about the association between telehealth utilization and clinical outcomes. Better understanding telehealth's limitations will allow us to optimize its usage, prevent poor clinical outcomes, and avoid widening existing disparities.

## AUTHOR CONTRIBUTIONS

**Armon Azizi:** Conceptualization (equal); data curation (lead); formal analysis (lead); investigation (equal); methodology (equal); project administration (equal); writing – original draft (equal); writing – review and editing (equal). **Aditya Mahadevan:** Conceptualization (equal); data curation (equal); investigation (equal); methodology (equal); project administration (equal); writing – original draft (lead); writing – review and editing (lead). **Jagmeet S. Arora:** Conceptualization (equal); data curation (equal); investigation (equal); methodology (equal); project administration (equal); writing – original draft (equal); writing – review and editing (equal). **Elaine Chiao:** Conceptualization (equal); investigation (equal); methodology (equal); project administration (equal); writing – original draft (equal); writing – review and editing (equal). **Sora Tanjasiri:** Conceptualization (equal); methodology (equal); project administration (equal); supervision (equal); writing – original draft (equal); writing – review and editing (equal). **Farshid Dayyani:** Conceptualization (equal); investigation (equal); methodology (equal); project administration (equal); supervision (lead); writing – original draft (equal); writing – review and editing (equal).

## FUNDING INFROMATION

The authors have no relevant financial disclosures. No funding was provided for this study.

## CONFLICT OF INTEREST STATEMENT

No authors declare no conflicts of interest.

## Supporting information


Figure S1.

## Data Availability

The datasets generated and analyzed for the study contain identifiable patient and visit data. Due to concerns regarding patient confidentiality and in adherence to HIPAA regulations, the data will not be publicly shared. Aggregate data may be shared upon request to the corresponding author.
