# Supplementary material for: Associations between language, telehealth, and clinical outcomes in patients with cancer during the COVID‐19 pandemic
Source: Cancer Med. 2024 Sep 23;13(18):e70099. doi: 10.1002/cam4.70099 (PMC11419674; doi:10.1002/cam4.70099)
Supplement: Supplementary file 1 — Figure S1. [file CAM4-13-e70099-s001.docx]

**Supplemental Figures Associated With: *Associations between Language, Telehealth, and Clinical Outcomes in Cancer Patients during COVID-19***

Authors: Armon Azizi, BS^1^*, Aditya Mahadevan, BS^1^*, Jagmeet S. Arora, BS^1^, Elaine Chiao, BS^1^, Sora Tanjasiri DrPh, MPH^2^, Farshid Dayyani, MD, PhD^3^**

*** These authors contributed equally**

**** Corresponding Author**

**Affiliations:**

1. School of Medicine, University of California Irvine, Irvine, CA, USA
2. Department of Health, Society & Behavior, Program of Public Health, University of California, Irvine, Irvine, CA, USA.
3. Division of Hematology/Oncology, University of California Irvine Health, Orange, CA, USA

**Supplemental Figure 1:**


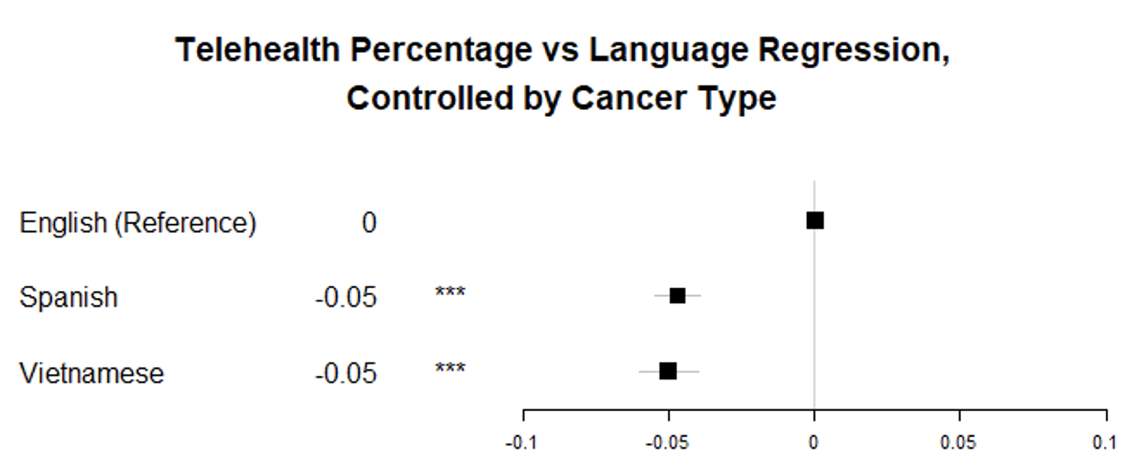


**Supplemental Figure 1:** Forest plot displaying the association between preferred language and telehealth utilization. Regression coefficients and significance are displayed. Analysis was performed using patient cancer type as covariate in the multivariate model.

**Supplemental Figure 2:**


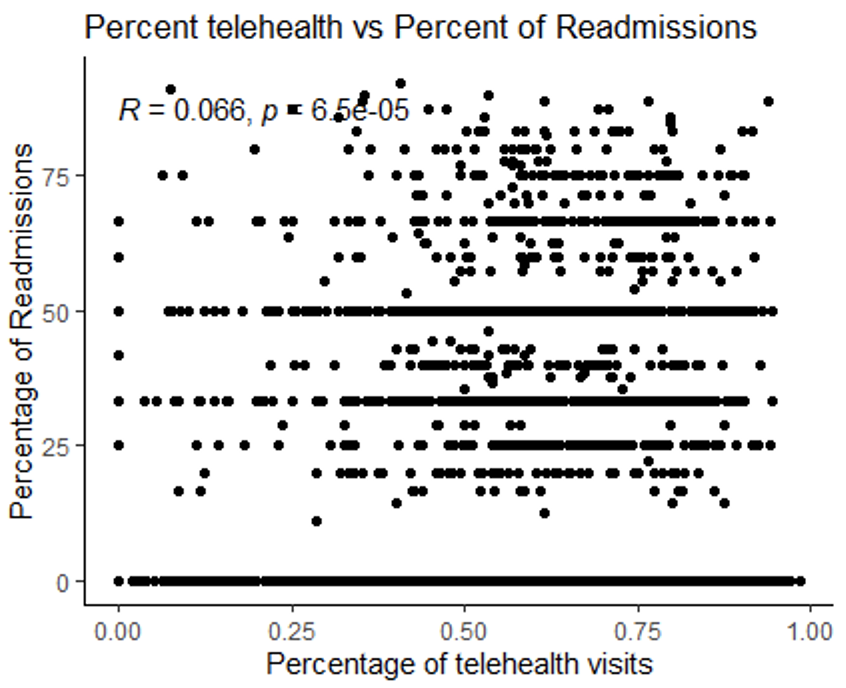


**Supplemental Figure 2:** Dotplot comparing the percentage of visits that were telehealth vs the fraction of admissions that were re-admissions (within 30 days of a discharge) across all patients in the study.

**Supplemental Figure 3:**


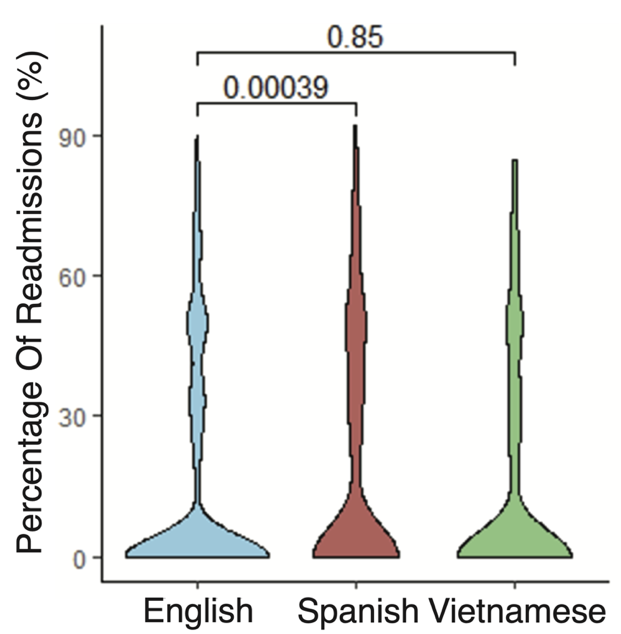


**Supplemental Figure 3:** Percentage of admissions that were re-admissions (within 30 days of a discharge) across the top 3 language groups in the study.
